# Comparison of robotic AI-assisted and manual pedicle screw fixation for treating thoracolumbar fractures: a retrospective controlled trial

**DOI:** 10.3389/fbioe.2025.1491775

**Published:** 2025-04-04

**Authors:** Xun Xiao, XingKun Wang, Bin Meng, Xin Pan, Hua Zhao

**Affiliations:** ^1^ Department of Orthopaedics, Qilu Hospital of Shandong University, Cheeloo College of Medicine, Shandong University, Jinan, Shandong, China; ^2^ Cheeloo College of Medicine, Shandong University, Jinan, Shandong, China

**Keywords:** robotic AI-assisted navigation, assisted screw placement, manual screw placement, thoracolumbar fractures, safety and efficacy

## Abstract

**Objective:**

To compare the clinical efficacy and screw placement accuracy of robot artificial intelligence (AI)-assisted percutaneous screw fixation and conventional C-arm-assisted percutaneous screw fixation (manual placement) in the treatment of thoracolumbar single-segment fractures without neurological symptoms.

**Methods:**

This study is a single-center retrospective analysis involving patients with thoracolumbar single-segment fractures without neurological symptoms. Patients were divided into Group A (robotic AI-assisted placement) and Group B (manual placement). Clinical outcomes such as operative time, intraoperative fluoroscopy frequency, screw placement accuracy, postoperative complications, length of hospital stay, and postoperative pain were compared between the two groups.

**Results:**

Group A showed significantly better screw placement accuracy, fewer intraoperative fluoroscopy attempts, shorter fluoroscopy time, and fewer guidewire adjustments compared to Group B (P < 0.05). Additionally, Group A had shorter hospital stays, a lower incidence of postoperative complications, and short-term greater improvement in Visual Analog Scale (VAS) scores (P < 0.05). However, after 1 year of follow-up, there was no statistically significant difference between the two groups in the improvement of VAS scores.

**Conclusion:**

Robotic AI-assisted placement improves pedicle screw placement accuracy, reduces intraoperative fluoroscopy frequency and time, alleviates postoperative pain, and accelerates patient recovery. This approach aligns with the principles of enhanced recovery in orthopedic surgery and holds promise for wider clinical application in the treatment of thoracolumbar fractures.

## 1 Introduction

The thoracolumbar region refers to the transitional zone between the thoracic and lumbar spine. Anatomically, the thoracic spine exhibits greater stability due to the structural reinforcement provided by the thoracic cage. In contrast, the lumbar spine demonstrates enhanced flexibility, attributed to the absence of rib constraints and the presence of thicker intervertebral discs (Wood et al., 2014). Furthermore, the abrupt change in the orientation of the facet joints, shifting from the coronal-horizontal position in the thoracic spine to the sagittal-vertical position in the lumbar spine, results in stress concentration at the thoracolumbar junction (Tomczyk-Warunek et al., 2024). This biomechanical transition renders this region particularly susceptible to traumatic injuries, accounting for 50%–60% of all thoracolumbar spinal fractures (Gertzbein, 1992). Currently, the primary clinical treatment for fractures in this region involves pedicle screw fixation. This approach aims to restore spinal stability by improving the percentage of anterior vertebral height, reducing the Cobb angle, and correcting kyphotic deformities (Zhang et al., 2022). Traditional surgical approaches include Open Screw Fixation (OSF) and Percutaneous Screw Fixation (PSF). Compared to OSF, PSF effectively reduces intraoperative bleeding and soft tissue damage. However, it relies on C-arm fluoroscopy, which increases radiation exposure and demands higher precision in the procedure (Yoshikawa et al., 2023; Peters et al., 2024).

In recent years, with the advancement of imaging equipment and computer-aided technology, various navigation technologies have emerged (Zhou et al., 2025). Compared to conventional navigation technologies, robotic guidance (RG) can overcome the limitations of human physiological fatigue, ensuring high operative accuracy, good repeatability, and strong operational stability (Shi et al., 2021; Kou et al., 2023). Benefiting from these advantages, it is widely used in pedicle screw insertion (Laine et al., 2000), joint replacement surgery (Ren et al., 2019), scoliosis correction surgery (Chen et al., 2020), and various minimally invasive surgeries (Wan et al., 2024). In the field of spinal surgery, RG has demonstrated excellent clinical outcomes (Kanna et al., 2021). Studies have shown that, compared to PSF, RG similarly reduces damage to soft tissues and paraspinal muscles. However, RG provides precise control over surgical instrument movement, minimizing intraoperative vibration and errors, and thus improving screw placement accuracy (Kam et al., 2019). Additionally, RG integrates preoperative and intraoperative imaging data, reducing the need for fluoroscopic imaging and lowering radiation exposure (Mason et al., 2014; Jiang et al., 2020). Currently, robot-assisted spinal surgical systems in common clinical use include: SpineAssist/Renaissance and Mazor X (Mazor Robotics, now Medtronic), ExcelsiusGPS (Globus Medical), Cirq (Brainlab), Rosa (Zimmer Biomet), SPINEBOT (CUREXO, South Korea), BITEBOT II (DAAI Robotics, China), and TiRobot (Beijing Tinavi Medical Technologies, China). (Sardi et al., 2023; Guiroy et al., 2023; Tian et al., 2020). The TiRobot system is a bone surgery robot independently developed in China, with complete self-owned intellectual property rights. It is the world’s first multi-indication bone navigation robot, capable of directly performing surgical path planning, detecting patient respiratory movements and instrument deviations, thereby significantly improving the precision and safety of surgery (Ma et al., 2023; Tian et al., 2019).

This study conducts a retrospective analysis to compare the clinical efficacy and safety of robotic-assisted pedicle screw placement using TiRobot versus manual percutaneous screw placement in the treatment of thoracolumbar fractures, providing theoretical support for the clinical application of orthopedic robots in thoracolumbar fracture treatment.

## 2 Methods

### 2.1 Study populations

This study retrospectively analyzed the clinical data of 50 patients with single-level thoracolumbar fractures without neurological symptoms, who were treated in the Department of Orthopedics at Qilu Hospital of Shandong University from 2018 to September 2022. The Load Sharing Classification (LSC) was evaluated in all patients. This system evaluates three key aspects of the fracture: the degree of vertebral body comminution, the degree of postoperative kyphosis correction, and the sagittal plane collapse of the vertebral body. Each factor was categorized into three grades, with scores ranging from 1 to 3 points (McCormack et al., 1994). Based on the surgical method, the patients were divided into two groups: Group A (robotic AI-assisted placement group, 20 cases) and Group B (manual screw placement group, 30 cases). The inclusion criteria were: (1) Preoperative imaging confirmed that the thoracolumbar fracture was limited to a single-level fracture at T11-L2; (2) The fracture occurred within 2 weeks and there was no spinal cord or nerve root injury; (3) AO classification of A1 or A3, with clear indications for spinal fixation surgery; (4) Complete and preserved clinical data with a follow-up period of ≥1 year; (5) All patients underwent short-segment pedicle screw fixation across the injured vertebra. Exclusion criteria included: (1) Patients with other spinal conditions, such as spinal deformities, spondylolisthesis, lumbar spinal stenosis, ankylosing spondylitis, etc.; (2) Patients with other severe trauma; (3) Patients who had previously undergone similar surgeries due to spinal conditions; (4) Patients with severe osteoporosis.

### 2.2 Surgical method

**FIGURE 1:**
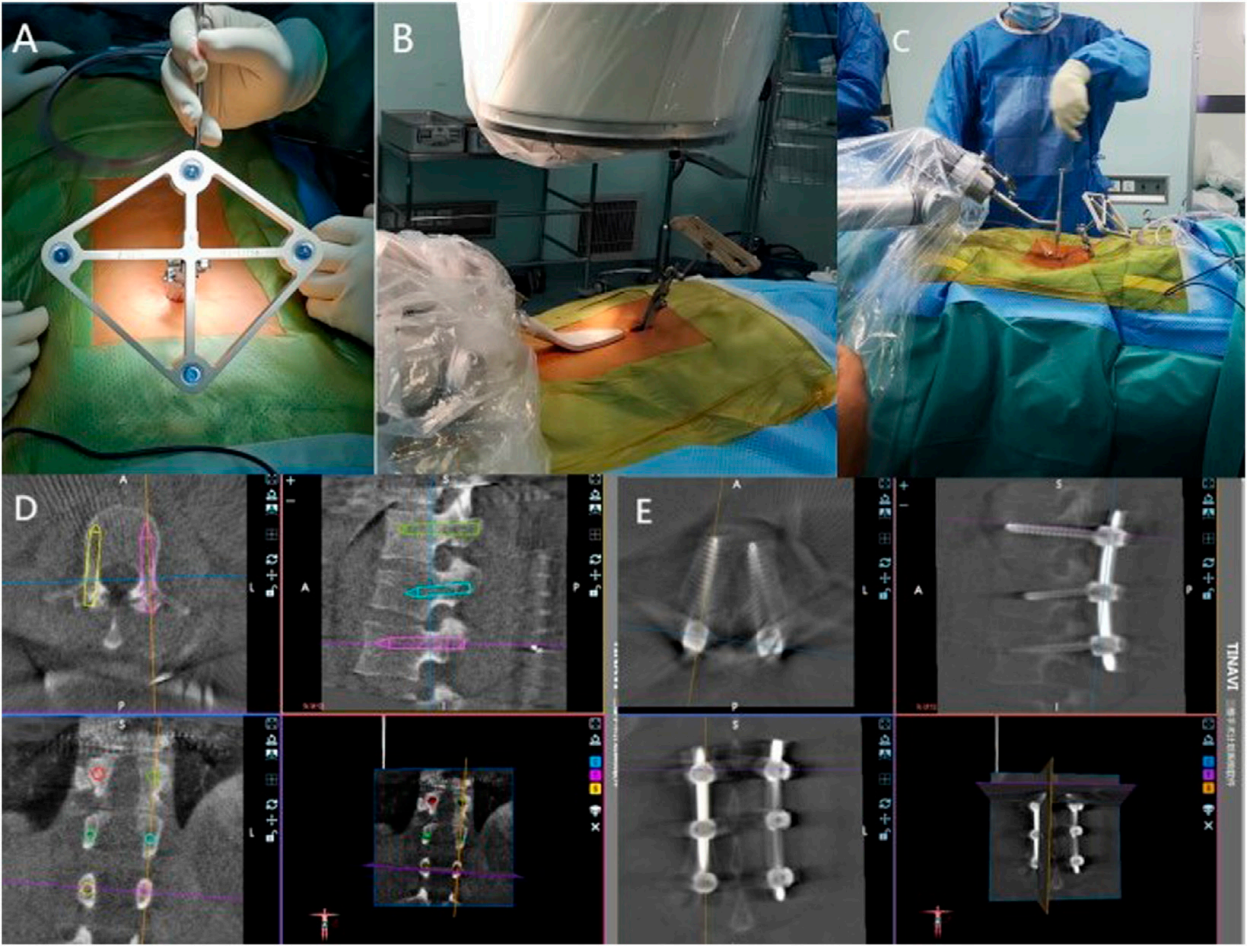
Screw insertion procedure using the TiRobot system. **(A)** A tracker for the spinal navigation was placed on a spinous process. **(B)** C-arm navigation. **(C)** K-wire was drilled into the vertebrae. **(D)** Preoperative planning and design are performed in the robotic workstation. **(E)** All screws were implanted.

In Group A, the TiRobot orthopedic robotic system was used to assist with percutaneous pedicle screw placement. The TiRobot was connected to the 3D-C arm system and calibrated. After standard preoperative preparations, a spinous process clamp and tracker were installed, the robotic arm was sterilized, and the guide device was set up. A 3D image was generated using the C-arm fluoroscopy system (with the surgeon outside the operating room during fluoroscopy). The surgical site images were displayed on the robotic workstation, and the entry point, direction, diameter, and length of the pedicle screws were planned. Once the robotic arm was positioned, the surgeon inserted the sleeve along the fixed trajectory of the robotic arm and made a vertical skin incision of 1.0–1.5 cm. After blunt dissection of the subcutaneous tissue, the sleeve was inserted into the guide device, and a guide wire was placed along the sleeve, followed by the insertion of pedicle screws along the guide wire. These steps were repeated for all screws according to the pre-planned trajectory (Figure 1). After confirmation of screw placement via C-arm fluoroscopy, a titanium rod was inserted, reduction was achieved using a distractor, the nuts were tightened, hemostasis was performed, the wound was irrigated, and the incision was sutured in layers, concluding the robotic procedure.

In Group B, patients underwent traditional percutaneous pedicle screw fixation and fracture reduction under C-arm fluoroscopic guidance. Under general anesthesia, C-arm fluoroscopy was used to locate and mark the surface projection of the pedicle. The skin incision site was confirmed and marked. After skin disinfection and draping, a vertical incision of approximately 1.5 cm was made along the marked line. A guidewire was inserted in line with the pedicle projection. After confirming the correct placement of the guidewire using C-arm fluoroscopy, the channel was dilated, and pedicle screws were inserted along the guidewire. This process was repeated for all screws. Once the correct placement of the screws was confirmed by C-arm fluoroscopy, a titanium rod was inserted, reduction was achieved using a distractor, and the nuts were tightened. The incision was irrigated several times with saline and then sutured in layers.

### 2.3 Postoperative management and efficacy observation indicators

Both groups received identical postoperative treatment, including wearing thoracolumbar braces. X-rays and CT scans were taken 3 days postoperatively, and all patients were followed up at 6 months and 1 year with thoracolumbar X-rays. The total follow-up period exceeded 1 year.

**FIGURE 2:**
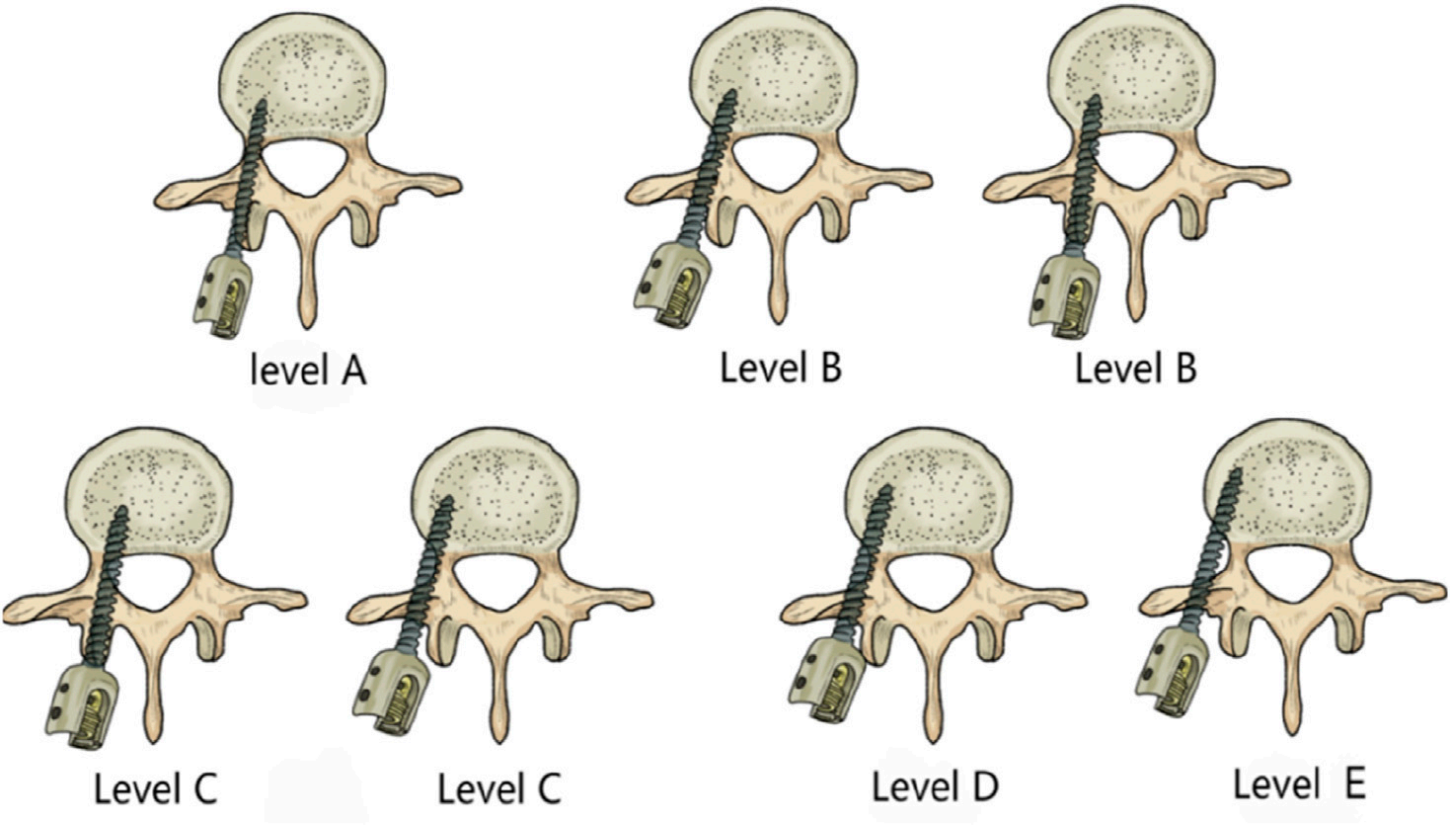
Grading of pedicle screw misplacement.

The following parameters were compared between the two groups: operative time, intraoperative blood loss, fluoroscopy frequency, sagittal Cobb angle, VAS score, quality of life score, and accuracy of pedicle screw placement. Pain intensity was assessed using the VAS before surgery, 3 days after surgery, and at 6 months and 1 year postoperatively. Patients rated their pain on a numeric scale from 0 to 10, where 0 represented no pain, 2-4 represented mild pain, 5-7 represented moderate pain, 8-9 represented severe pain, and 10 represented the most severe pain (Sun et al., 2020). Quality of life was assessed using the 36-Item Short Form Survey (SF-36). Patients rated their subjective experience in 8 dimensions, with scores ranging from 0 to 100 in each dimension. The raw scores were converted to standardized scores using the formula: standardized score = (raw score - minimum possible score)/(maximum possible score - minimum possible score) × 100%. The total SF-36 score was obtained by summing the standardized scores of all dimensions. Higher scores indicated a better quality of life. Operative time: In Group A, timing began from the initiation and calibration of the TiRobot until the completion of suturing and bandaging. In Group B, timing began from the start of skin disinfection and draping until the completion of suturing and bandaging. The accuracy of pedicle screw placement was evaluated using CT + 3D reconstruction. The degree of pedicle screw cortical breach was graded according to the Gertzbein-Robbins classification system, which includes five grades (Schizas et al., 2007): Grade A: Screw entirely within the pedicle; Grade B: Cortical breach <2 mm; Grade C: Cortical breach ≥2 mm but <4 mm; Grade D: Cortical breach ≥4 mm but <6 mm; Grade E: Cortical breach ≥6 mm. Screws graded as A and B were considered clinically acceptable (Figure 2).

### 2.4 Statistical methods

All data in this study were statistically analyzed using SPSS 25.0 (IBM Corp, Armonk, New York, USA). Normality of the data was assessed using the Shapiro-Wilk test. For continuous variables following a normal distribution, independent samples t-tests were used (expressed as Mean ± SD). For continuous variables not following a normal distribution, the Mann-Whitney U test was applied (expressed as median and interquartile range). Categorical variables were compared using the chi-square test. If any expected frequency in the contingency table was less than 5, Fisher’s exact test was used instead. A p-value of <0.05 was considered statistically significant (Yu et al., 2018).

## 3 Results

### 3.1 Baseline data analysis

The baseline data of the two groups of patients were collected and compared. The results showed that there were no significant differences between Group A and Group B in terms of gender, age, BMI,LSC and fracture segments (P > 0.05, Table 1).

**TABLE 1:** Baseline characteristics of the study participants.

Characteristic	Group A N = 20	Group B N = 30	*P*
Age (Years)	46.7 ± 14.0	47.4 ± 9.8	0.820
Gender (Female/Male)	5/15	11/19	0.386
BMI(kg/m^2^)	27.0 ± 6.2	24.5 ± 4.2	0.125
LSC	5.69 ± 0.81	5.73 ± 0.83	0.762
Injured vertebra [n (%)]			0.066
T12	6 (30.0)	13 (43.33)	
L1	9 (45.0)	16 (53.33)	
L2	5 (25.0)	1 (3.33)	

### 3.2 Comparison of intraoperative data between the two groups

**FIGURE 3:**
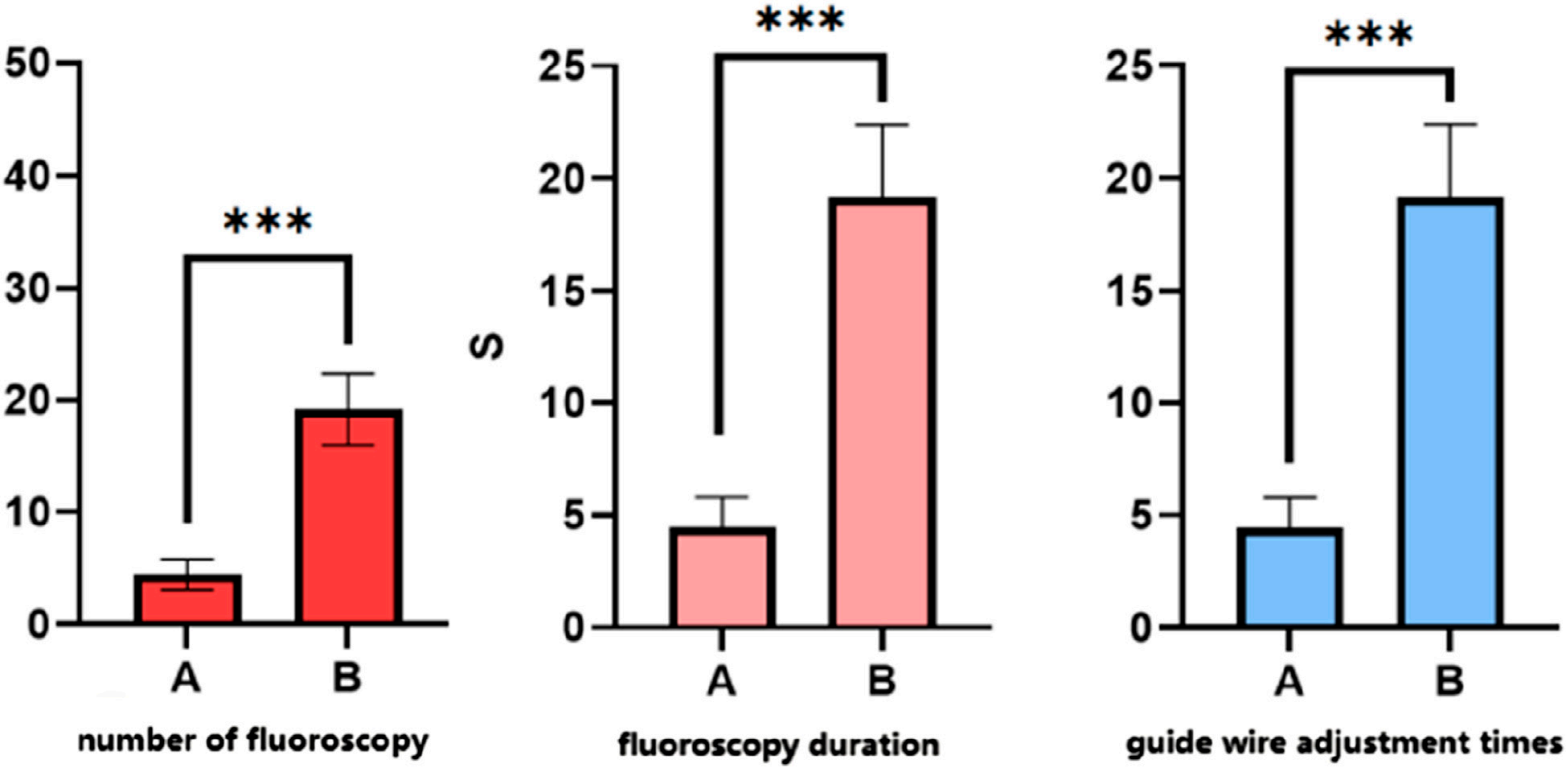
Difference in the number of fluoroscopies, fluoroscopy time,and guidewire adjustments between the two groups. Note: ***P < 0.001.

As shown in Table 2,there was no statistically significant difference in intraoperative blood loss between the two groups [50 (30, 150) mL vs 50 (50, 100) mL] (P > 0.05). However, the surgery duration was significantly longer in Group A than in Group B [145 (135, 170) minutes vs 120 (110, 145) minutes], and this difference was statistically significant (P < 0.05). Figure 3 shows that group A had fewer fluoroscopy attempts [5 (5, 6) vs 27 (23, 34)], shorter fluoroscopy duration [10 (9, 11) seconds vs. 36 (31, 41) seconds], and fewer guidewire adjustments [5 (3, 5) vs 18 (17, 21)].

**TABLE 2:** Comparison of intraoperative observation indicators between the two groups.

Characteristic	Group A N = 20	Group B N = 30	P
Surgery duration (min)	145 (135,170)	120 (110,145)	0.029
Intraoperative blood loss (ml)	50 (30,150)	50 (50,100)	0.822

### 3.3 Analysis of VAS score, sagittal cobb angle, and PAHC in both group

At the 3-day and 6-month postoperative follow-ups, Group A demonstrated significant improvements in VAS scores (P < 0.05). However, no statistically significant differences were observed between the two groups in preoperative assessments or at the final follow-up. Additionally, no significant differences in Cobb angle measurements were noted between the groups throughout the study period (Table 3).

**TABLE 3:** Analysis of VAS score, sagittal Cobb angle, PAHC.

Characteristic	Group A N = 20	Group B N = 30	P
Cobb (°)			
preoperative surgery	15.83 ± 7.63	15.22 ± 7.17	0.780
3 days postoperative	11.51 ± 6.14	11.44 ± 6.15	0.971
6 months postoperative	12.21 ± 6.10	11.57 ± 5.79	0.719
1 year postoperative	12.82 ± 5.81	12.94 ± 5.06	0.936
VAS			
preoperative surgery	6.43 ± 0.90	6.33 ± 0.87	0.712
3 days postoperative	3.33 ± 0.94	5.47 ± 0.85	<0.001
6 months postoperative	2.19 ± 0.59	3.53 ± 0.85	<0.001
1 year postoperative	0.21 ± 0.16	0.27 ± 0.12	0.073

### 3.4 Comparison of screw placement accuracy between the two groups

**FIGURE 4:**
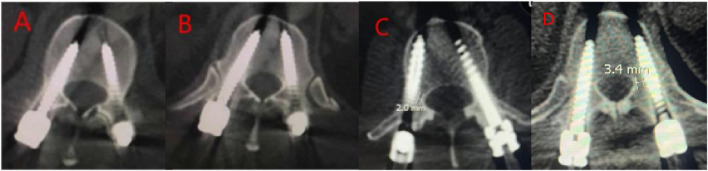
Screw placement in Group A. **(A, B)** Grade A screws; **(C)** Grade B screw; **(D)** Grade C screw.

**FIGURE 5:**
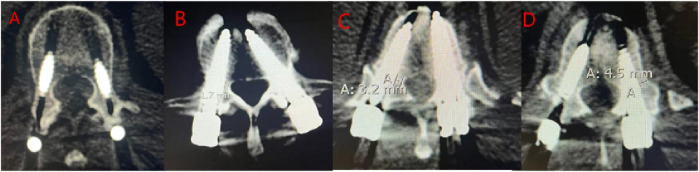
Screw placement in Group B. **(A)** Grade A screws; **(B)** Grade B screws; **(C)** Grade C screws; **(D)** Grade D screws.

This study compared the accuracy of screw placement between the two groups. The results showed that out of 120 screws placed in Group A, 103 were classified as Grade A, 12 as Grade B, and 5 as Grade C. In Group B, out of 160 screws, 109 were classified as Grade A, 34 as Grade B, 16 as Grade C, and 1 as Grade D (Table 4; Figures 4, 5). Group A had a higher proportion of Grade A screws compared to Group B (85.83% vs 68.13%). Additionally, Group A had a higher number of clinically acceptable screws (Grade A and B) than Group B (95.83% vs. 89.38%). The differences between the two groups were statistically significant (P < 0.05).

**TABLE 4:** Comparison of screw placement accuracy between the two groups.

Characteristic	Group A N = 20	Group B N = 30	P
Screws numbers	120	160	0.029
Grade			
A	85.83%	68.13%	<0.001
B	10.00%	21.25%	
A + B	95.83%	89.38%	<0.001
C	4.17%	10.00%	
D	0	0.62%	
E	0	0	

### 3.5 Comparison of postoperative data between the two groups

Compared to Group B, Group A had a shorter hospital stay and significantly higher survival scores. The differences were statistically significant (P < 0.05, Table 5). Additionally, group A had a lower incidence of postoperative complications, including hypostatic pneumonia, pressure ulcers, urinary tract infections, wound infections, and screw breakage. The differences between the two groups were statistically significant (P < 0.05, Table 6).

**TABLE 5:** Comparison of the length of hospital stay and survival scores between the two groups.

Characteristic	Group A N = 20	Group B N = 30	P
Length of stay (Day)	5.62 ± 0.65	7.83 ± 0.69	<0.001
survival scores	82.33 ± 3.03	72.17 ± 2.68	<0.001

**TABLE 6:** Comparison of postoperative complications between the two patient groups.

Postoperative complications	Group A N = 20	Group B N = 30	P
Hypostatic pneumonia	0	1	
Pressure sores	0	3	
Urinary tract infection	0	2	
Wound infection	2	1	
The screw fracture	1	2	
Total	3	9	<0.001

## 4 Discussion

**FIGURE 6:**
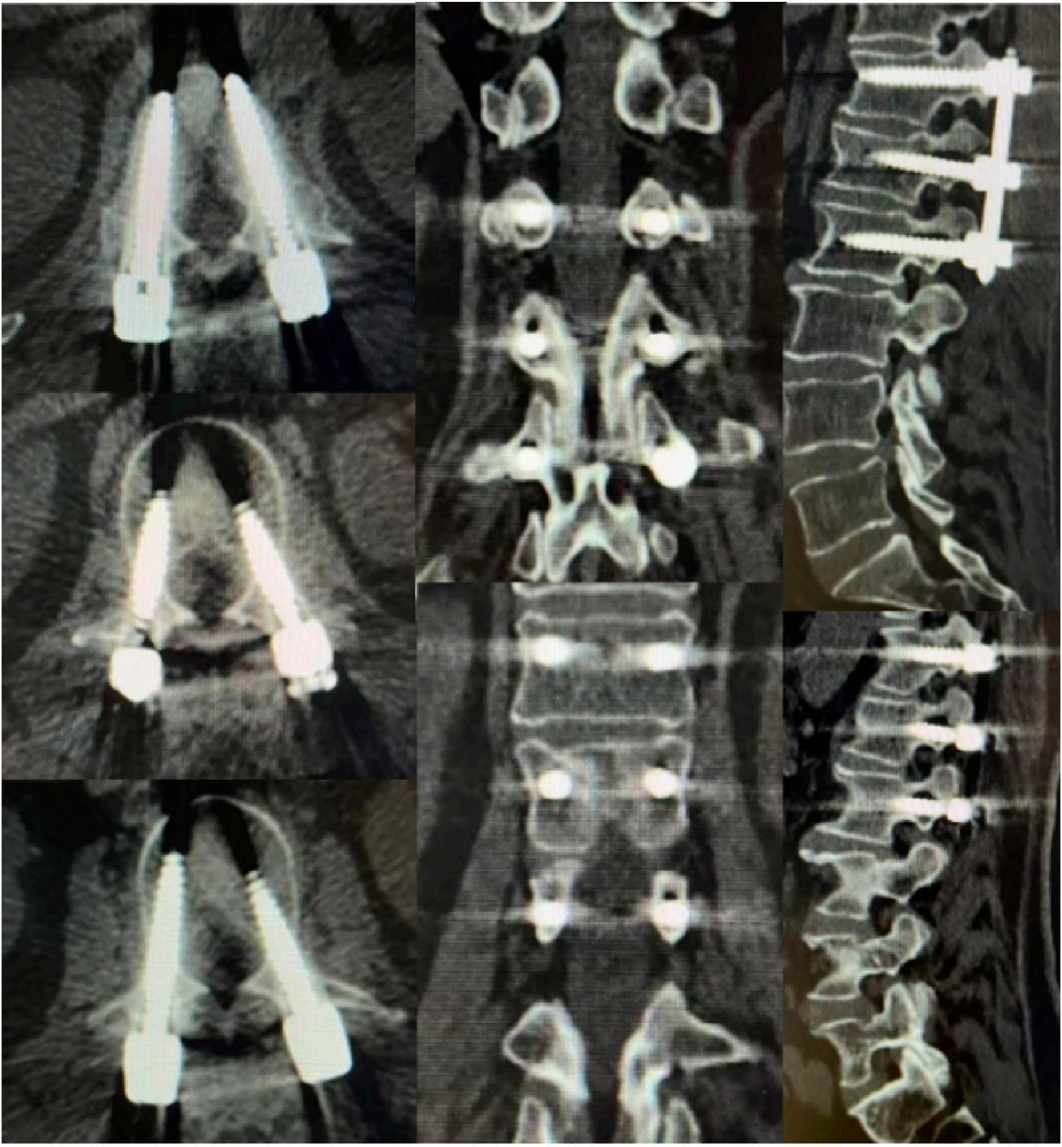
A 5-year-old female patient underwent robotic-assisted percutaneous pedicle screw fixation for L1 fracture reduction. All screws were classified as grade A screws based on imaging.

In recent years, robot-assisted pedicle screw placement technology has rapidly developed, with increasing clinical studies indicating that robotic-assisted techniques surpass traditional manual operations in terms of screw placement accuracy (Wang et al., 2024). A randomized controlled trial by Feng et al. (2019) demonstrated that 98.5% of the screws in robot-assisted pedicle screw placement achieved Grade A positioning. In this study, we found that the acceptable screw rate in Group A was 95.87%, significantly higher than that in Group B (89.38%) (Figure 6). This could be attributed to the preoperative planning using 3D imaging, the mechanical stability, and the precise control capabilities of the robotic arm, as well as the real-time tracking of slight patient movements and respiratory motions during surgery by the spinal robotic system (Han et al., 2019).

In clinical practice, The Cobb angle is used not only to evaluate the degree of spinal curvature but also to assess vertebral collapse and kyphotic correction. Polly et al. (1996), after measuring lateral X-rays of 60 complete spines, found the Cobb angle measurement to be more reliable than lumbar lordosis measurement. Therefore, in this study, Cobb angles were evaluated in both groups preoperatively, 3 days postoperatively, at the first postoperative follow-up, and at the final follow-up.

**FIGURE 7:**
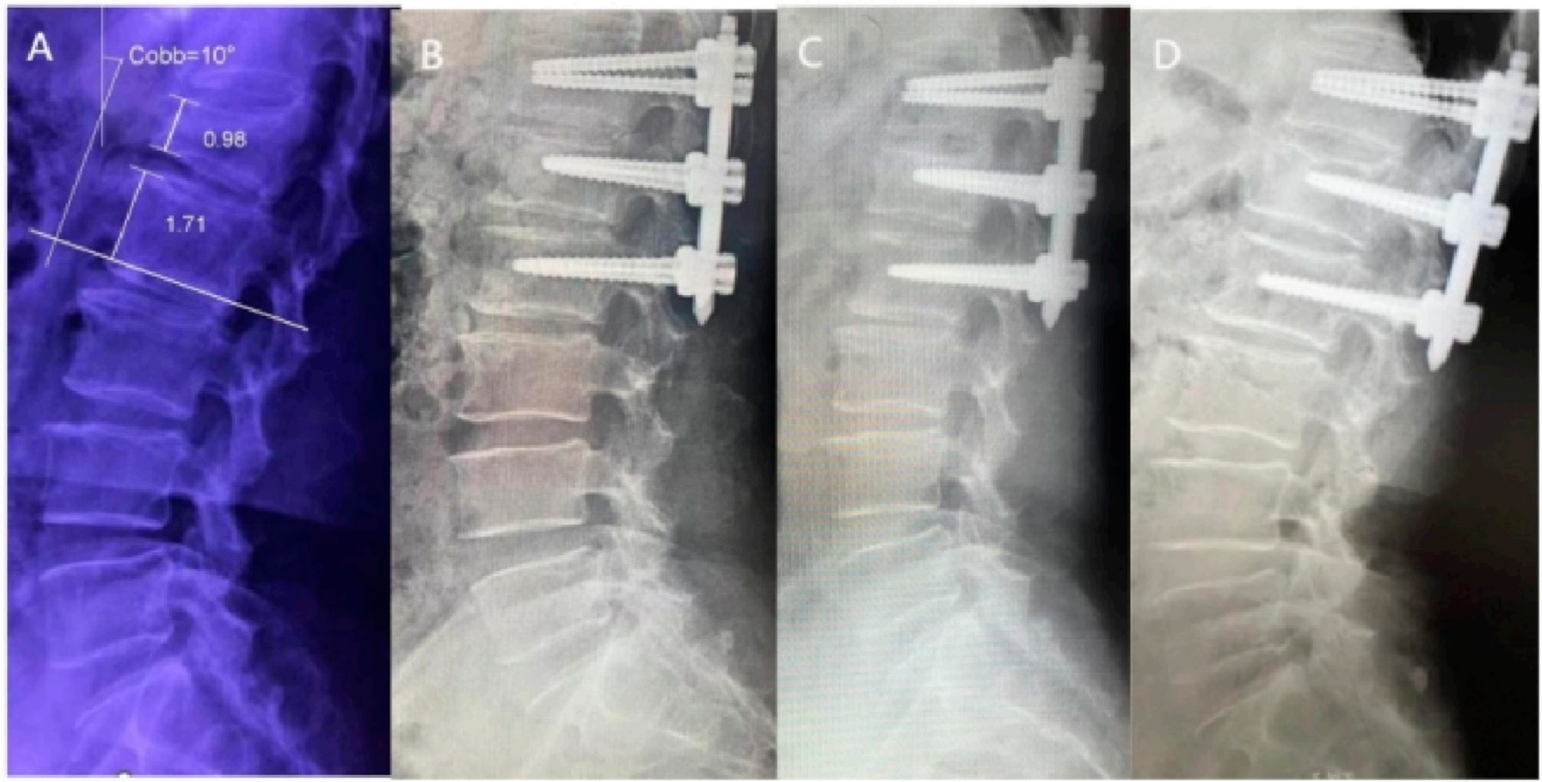
X -rays of a 55-year-old female patient treated with robotic-assisted percutaneous pedicle screw fixation. **(A)** Preoperative; **(B)** 3 days postoperative; **(C)** 6 months postoperative; **(D)** 1 year postoperative.

**FIGURE 8:**
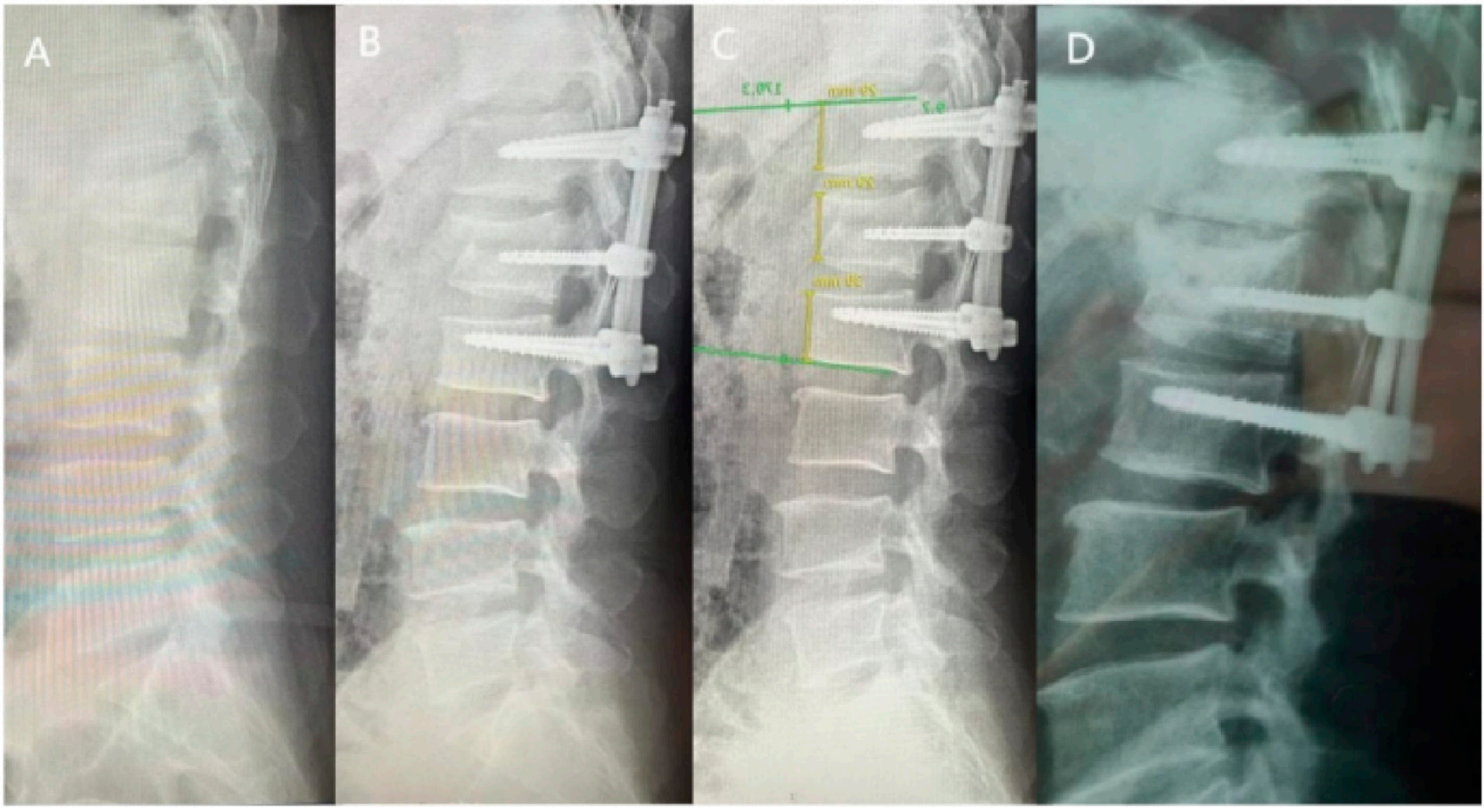
X-rays of a 69-year-old female patient treated with manual percutaneous pedicle screw fixation. **(A)** Preoperative; **(B)** 3 days postoperative; **(C)** 6 months postoperative; **(D)** 1 year postoperative.

The results demonstrated that no significant differences in Cobb angle measurements were observed between the groups throughout the study period (P > 0.05, Figures 7, 8), indicating comparable clinical efficacy between robot-assisted surgery and the manual group in fracture reduction. Notably, the freehand pedicle screw placement in this study was performed by a highly experienced spinal surgeon with nearly 30 years of clinical practice. The fact that the robot-assisted group achieved equivalent reduction outcomes fully validates the reliability of robot-assisted screw placement technology. Compared to the manual group, the robot-assisted group exhibited significantly lower Visual Analog Scale (VAS) scores at both the 3-day and 6-month postoperative follow-ups, suggesting a distinct advantage of robotic assistance in alleviating early postoperative pain. These differences may be attributed to the implementation of a preoperative three-dimensional planning system and intraoperative precision maneuvers, which enhance screw placement accuracy, minimize iatrogenic trauma to normal tissues, and thereby promote more substantial early postoperative recovery. However, the disparity in pain scores between the two groups disappeared by the final follow-up, demonstrating that both techniques ultimately restore spinal stability and biomechanical integrity through osseous healing. Another noteworthy aspect is that, compared to conventional manual surgery, robot-assisted surgery involves fewer fluoroscopic views and shorter fluoroscopy times. Robotic technology can reduce reliance on intraoperative fluoroscopy, allowing surgeons to leave the operating room during 3D imaging and enabling the removal of the C-arm after preoperative planning, thereby limiting radiation exposure for surgical teams (Han et al., 2019). This advantage is particularly valuable for young and less-experienced surgeons, as studies have shown that surgeons with insufficient experience may require more frequent fluoroscopic checks during manual surgeries (Peng et al., 2020). Additionally, the short-term advantages of robotic surgery, such as reduced fluoroscopy time, lower VAS scores, fewer complications, and shorter hospital stays, alleviate both physical and financial burdens on patients (Peng et al., 2020), aligning with the principles of Enhanced Recovery After Surgery (ERAS).

Despite the significant advantages of robot-assisted surgery in the early postoperative period, the longer surgical times remain a concern worth noting. This study found that the operation duration for the RG group was longer than that for the manual group. The main reasons include: Firstly, the longer preoperative preparation time for robot-assisted surgery (Elswick et al., 2020); secondly, the relatively steep learning curve for robotic technology, as the surgeon’s level of proficiency directly affects surgical duration. However, as surgeons become increasingly familiar with this emerging technology, their operations are likely to become more efficient, potentially leading to shorter surgical times (Li et al., 2023). Additionally, the high equipment costs and limited cost-effectiveness of robotic surgeries are major barriers to their widespread adoption (Li et al., 2023). Many studies have reported that, compared to traditional techniques, robots provide minimal improvements while their associated costs are significantly higher (Yu et al., 2018; Gao et al., 2018). However, upon analysis, it was found that although the direct costs of robotic-assisted surgeries are higher, the reductions in hospital stay duration and postoperative complications also lead to lower overall healthcare expenditures (Peng et al., 2020). One study demonstrated that, over the course of a year, robotic technology saved a hospital $608,546 b y performing 557 elective spine surgeries (Menger et al., 2018). Furthermore, expanding the applications of robotic technology is also an effective way to enhance its cost-effectiveness. TiRobot can be applied to various anatomical locations for both open and minimally invasive surgeries, significantly broadening its range of applications and thereby reducing maintenance costs^29^. Despite the current challenges associated with robotic surgery, as the technology progresses, instrumentation will be refined and software systems will be upgraded. An increasing number of issues will be addressed, and the inherent advantages of robotic systems, such as high precision, reproducibility, and flexibility, are likely to become more apparent (Liu et al., 2016).

### 4.1 Limitations and weaknesses

This study has several limitations. First, it only included patients with single-level thoracolumbar fractures without neurological symptoms and AO type A fractures, limiting its generalizability and failing to explore robot-assisted screw placement in other fracture types or multi-segment fractures. Additionally, the study only assessed the accuracy of robot-assisted pedicle screw placement, without analyzing the learning curve or cost-effectiveness, which could affect its broader clinical adoption. The sample size was small (50 patients), potentially impacting statistical stability. The retrospective design may introduce selection bias and data limitations. Future research should expand the sample size, cover more fracture types, adopt prospective RCT designs, and conduct multi-center studies to enhance credibility and generalizability.

## 5 Conclusion

This retrospective analysis compared the clinical efficacy of TiRobot assisted percutaneous screw fixation with manual screw placement for the treatment of thoracolumbar single-segment fractures without neurological symptoms. The results indicate that robot-assisted screw placement offers superior accuracy, reducing the need for repeated guidewire adjustments, punctures, and screw-related pedicle damage. It also decreased surgery-related complications, significantly lowering patients’ early postoperative VAS scores and hospital stay duration, thereby achieving outstanding advantages in the early postoperative period. Additionally, it minimizes the number of fluoroscopic exposures and reduces fluoroscopy time, thereby lowering radiation exposure for both surgeons and patients. Compared to manual techniques, robot-assisted percutaneous screw placement demonstrates better accuracy, safety, and stability, making it a valuable option with significant clinical potential for widespread use.

## Data Availability

The raw data supporting the conclusions of this article will be made available by the authors, without undue reservation.
